# Human enteroviruses are not the cause of neurological impairments in children at the Korle-Bu Teaching Hospital

**DOI:** 10.11604/pamj.2014.18.232.3253

**Published:** 2014-07-21

**Authors:** Prudence Tettey, Ebenezer Badoe, Theophilus Adiku, Eva Obodai, John Kofi Odoom

**Affiliations:** 1 Department of Microbiology, University of Ghana Medical School, Korle-Bu, Accra, Ghana; 2 Department of Child Health, University of Ghana Medical School, Korle-Bu, Accra, Ghana; 3 Department of Virology, Noguchi Memorial Institute for Medical Research, University of Ghana, Legon, Accra, Ghana

**Keywords:** Convulsion, neurological, Human enterovirus, arbovirus, bacteriological, parasitological

## Abstract

**Introduction:**

Convulsions associated with fever and acute onset of unknown aetiology with case fatalities have become a long observed medical condition at the Child Health Department of the Korle-Bu Teaching Hospital. Children admitted to the department with seizures of undetermined origin and fever has been a source of diagnostic confusion. Studies from the Asia Pacific region suggest a link with non-polio enteroviruses. The aim of the study was to investigate the association between non-polio enterovirus and acute encephalopathy causing neurological morbidity in children.

**Methods:**

One hundred and fifty cerebrospinal fluid (CSF), throat swab and serum samples were collected from participants at the Child Health Department of the Korle-Bu Teaching Hospital for virus isolation and characterization. Samples were cultured on cells and positive culture assayed by microneutralisation. Direct PCR as well as multiplex PCR were used to detect other viral agents present.

**Results:**

Enterovirus isolation rate was approximately 0.67%. Intratypic differentiation by molecular characterization identified a poliovirus from vaccine origin. Further screening by real-time RT-PCR identified the virus as normal Sabin and not vaccine-derive poliovirus. No arbovirus was however detected.

**Conclusion:**

Non-polio enteroviruses and chikugunya virus were found not to be the etiologic agent responsible for the convulsion with neurologic morbidity observed in the Ghanaian children. Investigation for other viral agents is recommended.

## Introduction

Convulsion with acute onset in general is a common cause of admission in paediatric emergency wards and risk for neurological, cognitive impairment and epilepsy. Early diagnosis for the etiological cause and immediate clinical management is crucial to the survival of the child [1–4]. Clinical and experimental data suggest that prolonged seizures can have immediate and long-term adverse consequences on the immature and developing brain [5]. It is estimated that about 4% to 6% of all children will have a seizure in the first 16 years of life [6]. The incidence is predominant in children under the age of 3 years, with a declining frequency in older children [7]. Epidemiologic investigations have revealed that approximately 150,000 children will sustain a first-time, unprovoked seizure each year, and of those, 30,000 will develop epilepsy5 with the highest risk being among children with prior condition of neurodevelopmental abnormality and family history of afebrile seizures [6, 8]. The incidence of convulsions in developing countries including Ghana is higher than developed countries because of high infection rates [9–12]. Convulsion denotes a clinical symptom of an underlying pathologic condition with many possible causes. Convulsion may be caused by genetic and metabolic factors, fever, head injury, excessive alcohol intake, ischaemic stroke, intracranial haemorrhage, use of illicit drugs, meningitis, encephalitis and infection with parasites, bacteria or viruses [9, 13, 14]. Some of the viruses implicated in cases of convulsion include Human herpesvirus 6 [15, 16], influenza A [17, 18], Chikungunya virus [19, 20] and Human enterovirus 71 [21, 22].

At the Child Health Department of the Korle-Bu Teaching Hospital (KBTH), sporadic cases of convulsion with associated fever of unknown aetiology have become a long observed medical condition with diagnostic confusion. Routine parasitological and bacteriological investigations conducted have been inconclusive and no further investigations have been able to establish the cause of the disease. Cases include children between the ages of one day and twelve years with presentations of convulsion and fever, with occasional rashes. Many of the patients without any history of neurological problem were found to have developed temporal or permanent neurological impairment. Isolation of the causative agent would help to curtail unnecessary investigations, rationalise treatment, improve reliability of prognosis and prevent overuse of antimicrobial agents with consequent antimicrobial resistance.

In the Asian-Pacific regions, this manifestation of childhood convulsion associated with fever and neurological complication observed at the Child Health Department of the KBTH is usually associated with the non-polio enterovirus known as Human enterovirus 71 (HEV71) [23–27]. Currently, there is very little literature supporting the circulation of HEV71 in Africa which include the isolation of HEV71- like virus from children with acute flaccid paralysis in Central Africa Republic [28] and two small institutional outbreaks of HEV71 infection in HIV orphanages in Nairobi, Kenya [29]. However, the circulation of other enteroviruses is prevalent, which include poliovirus, Coxsackievirus, echovirus, hepatitis A virus and enterovirus 70 (5) [30, 31]. Although HEV71 is yet to be isolated in Ghana, migration, travel, tourism and pilgrimage of Muslims from Ghana to HEV71 endemic regions, may get infected and become a source of infection for others.

Epidemics of viral infections causing central nervous system effects are continuously being reported from around the world and clinicians are challenged to be abreast with local epidemiology. This study therefore aimed to investigate whether non-polio enterovirus was the etiological cause of the neurological disorders observed in the children.

## Methods

**Study population:** The study population comprised children between the ages of one day and twelve years old admitted to the hospital having clinical diagnosis of convulsion associated with fever and rash. Convulsions includes seizures lasting for at least half an hour, or convulsions followed by coma lasting two hours or more or convulsions followed by paralysis or other neurological signs not previously present and lasting 24 hours or more and convulsions that presented as encephalitis [32–34]. Only children with fever, convulsion, skin rash, herpangina, viral meningitis, viral encephalitis and other neurological manifestations were considered for the study.

**Virus isolation:** Viruses in stool specimens were isolated on Hep-2C (derived from human carcinoma cells) and RD (Rhabdomyosarcoma) cell lines in accordance with standard protocols (WHO,2004). Briefly, Hep-2C and RD cells were seeded in tissue culture tubes with growth medium (Eagle's MEM supplemented with 10% FCS) 48 hr prior to inoculation. Suspensions of faecal samples pre-treated with chloroform was inoculated on serum-free medium and incubated at 37oC and observed daily for the characteristic enterovirus cytopathic effect (CPE). The tubes with CPE up to 75% and above were harvested and kept at -20oC while those negative after 5 days of incubation were re-passaged (blind passage) on the same cell line and if it remained negative after 5 days was considered negative

**Microneutralization and real time PCR:** Identification of isolates on Hep-2C and RD cells lines was carried out by microneutralisation technique using polyclonal antisera raised in horse against Coxsackie and echoviruses prepared by the National Institute of Public Health and the Environment (RIVM), Netherlands. Microneutralisation assay with HEV71 antiserum was carried out on the isolates that were untypable with the antiseraum pools. Samples that showed neutralization in the wells with polio pool antisera selected for Real-Time Reverse Transcriptase polymerase Chain Reaction (rRT-PCR).

**RNA extraction and PCR:** Viral RNA was extracted by QIAamp Viral Mini Spin Protocol (Second edition, December 2005) according to manufacturer's instruction. Reverse Transcriptase Polymerase Chan Reaction (RT-PCR) with Pan-EV primers MD90 (5’- ATT GTC ACC ATA AGC AGC CA-3’) and anti-sense MD91 (5’ - CCT CCG GCC CCT GAA TGC GGC TAA T -3’) were used to amplify the VP1 region under the following conditions RT: 97°C for 3 minutes, PCR: 95°C for 45 seconds, 55°C for 45 seconds, and 70°C for 45 seconds and cooled to 4°C. The products were observed on 1% agarose gel electrophoresis.

**Data analysis:** Data analysis was done using SPSS version 19. The analysis involves frequency distribution of responses and cross tabulation of variables. The analyzed information was presented using tables, graphs, charts and other diagrams that depicted the pattern of findings.

**Ethical issues:** Ethical approval was obtained from the Ethical Committee of the College of Health Sciences, and Research Committee of the University of Ghana Medical School (UGMS). Informed consent was sought from parent/guardian of subjects before the commencement of the study. All ethical considerations were adhered to. Data collected from the study was handled anonymously and confidentially. Samples had only the identification numbers of the subjects to ensure anonymity. We protected the confidentiality of patients through use of codes.

## Results

**Demographic/clinical findings:** Eighty-two subjects were recruited for the study, with 49 (59.8%) being males. Majority of the subjects (67%) were between the ages of one day old and four years old. Apart from three of the subjects in junior high, the rest were either in primary school, kindergarten or not in school. According to the study, mothers were the usual care givers of children at home and were those attending to the children at the hospital. The educational status of the guardians showed that 14.6% of them did not have formal education. Of the remaining, 32.9% of them attended primary school, 26.8% junior high, 15.8% senior high and 9.7% had tertiary education.

**Figure 1:**
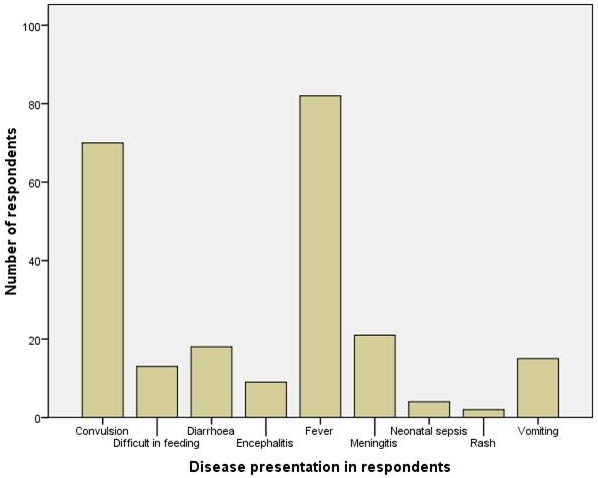
Clinical presentations of subjects recruited for the study.

**Figure 2:**
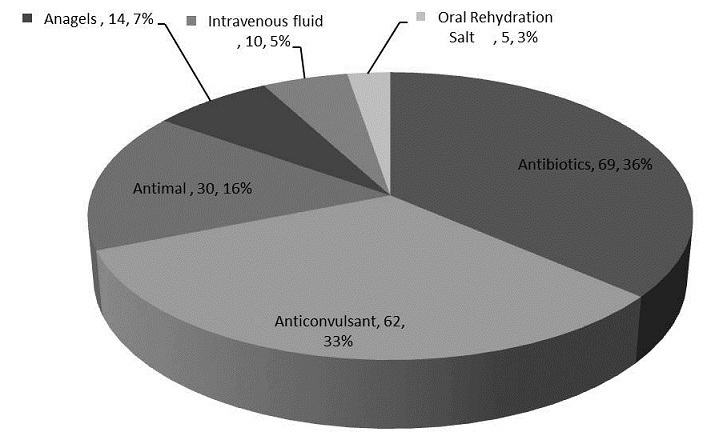
Medications administered to subjects recruited for the study

The clinical presentations as shown in Figure 1 indicates that the most frequent symptoms recorded in decreasing order were fever (100%), convulsion (85%), meningitis (25%), diarrhoea (22%), vomiting (18%), difficult in feeding (15.8%), encephalitis (11%), neonatal sepsis (4.8%) and with regards to the drugs that were administered, antibiotics, anticonvulsants and antimalarial drugs were the most frequently dispensed (Figure 2). Seventy-six (92.7%) of the subjects were given antibiotics, 52 (63.4%) anticonvulsants and 53 (44%) antimalarial drugs. Analgesics, intravenous fluid and Oral Rehydration Salt were given to 41.5%, 12% and 6.1% of the subjects respective.

**Figure 3:**
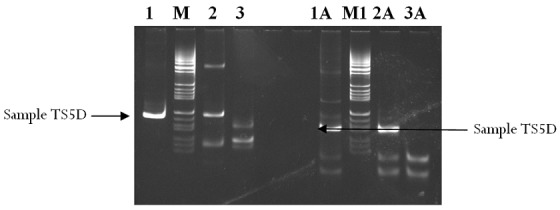
Electrophoretic patterns of pan-enterovirus (PEV) and pan-poliovirus (PPV): Lane 1, sample TS5D positive for pan-enterovirus; lane 1A, sample TS5D positive for pan-poliovirus. Lane M and M1, markers for PEV and PPV respectively; lane 2 and 2A, positive controls for PEV and PPV respectively; lane 3 and 3A, negative control for PEV and PPV respectively

**Virus isolation and characterisation:** Of the 150 samples, only one (0.7%) sample showed growth on Hep-2C and RD cell lines by the second day post inoculation. The positive sample was serotyped using pools of antisera to determine the serotype of virus present. No neutralization was observed in the wells containing the non-polio enterovirus antisera apart from those containing the polio pool, an indication of poliovirus present in the sample. Conventional PCR (Figure 3) and real-time RT-PCR performed on the isolate using Sabin specific primers used for intratypic differentiation in the WHO Polio Regional Reference Laboratory, NMIMR revealed the sample to be Sabin poliovirus type 1.

**PCR and multi-virus RT-PCR:** When RNA of all samples were extracted by Trizol method and amplified with standard primers, no sample was found positive. Samples screened for chikungunya virus using multi-virus RT-PCR showed no amplification.

## Discussion

The etiological cause of convulsion with acute onset in children in admission at the Korle-Bu Teaching Hospital was investigated for viral involvement such as non-polio enteroviruses and chikugunya virus using virus isolation and polymerase chain reaction techniques. The results as shown by the study did not link the viruses to the cause of the convulsions. Patients were managed with anticonvulsants, antibiotics, antimalarial and antivirals which were combined with Paracetamol, intravenous fluid (IVF) and oral rehydration salt (ORS) depending on the clinical presentation. The anticonvulsants were administered to manage clinically severe convulsion cases while antibiotics and antimalarial drugs were prescribed due to the febrile condition which may have either bacterial or parasitological origin.

Routine bacteriological and parasitological laboratory tests requested by the physicians to isolate the aetiological agent failed to detect neither bacteria nor any parasite. The failure of the laboratory to isolate the aetiological agent compelled these emergency physicians to continue the symptomatic management. In spite of this necessity, the practise of treating patients symptomatically is not always recommended because it may mask the presence of the underlying etiology which will then be forgotten or treated with great delay. Symptomatic treatment with antimicrobial agent can lead to antimicrobial resistance [35, 36]. The absence of parasites and bacteria during the laboratory investigation did not justify the extensive usage of the antimicrobial agents. While the hospital emergency department denotes a place of initiating empiric antimicrobial therapy as a form of medical intervention and also a site of extensive use of antimicrobial agents, emergency clinicians and all other clinicians must be fully conscious of the fact that inappropriate use and over prescribing of antimicrobial agents accelerates the development of antimicrobial resistance [37, 38]. Antimicrobial resistance, as it is already known, inflates the patient's budget, prolonging stay in hospital and also pressurizes drug manufacturing companies to make available new drugs that these agents would be susceptible to [39, 40].

To many emergency room physicians, the threat of antimicrobial resistance has not sunk in yet. It has been apparent through this study that emergency department physician's fundamental and principal concern in an emergency situation is how possible he could resuscitate his patient and that, the issue of the impact of antimicrobial use on the prevalence of resistance was not a crucial consideration at that moment. Many physicians and patients do not see antimicrobial resistance as a reason to abstain from its use [41, 42]. Emergency department physicians may therefore not be different from other physicians in their frequent prescription of antimicrobials for conditions that do not appear to profit from their use. This raises a general concern if the principle of prudent use of antimicrobials is being adhered to.

The findings from this study could not establish non-polio enterovirus or chikugunya association with the etiological cause of the manifestation. All molecular virological assays to determine the aetiology after RNA extraction from 150 samples yielded only one positive for poliovirus. Further characterization identified the poliovirus as a Sabin 1 poliovirus. The virus was obtained from the throat swab of a one month old baby with the presentations of fever, convulsion and fast breathing. These symptoms are not characteristic of poliovirus infection. Since Sabin 1 poliovirus is a component of the oral polio vaccine (OPV), the child might have acquired the virus from either polio vaccination during the recent national polio immunization days, contact with immunized person or from the environment. The oral polio vaccine (OPV) apart from seeding the gut of the recipient could also immunize children in contact with the faeces of the OPV recipient [43, 44]. It is also a way of gaining natural immunity when a person has not received the OPV. The OPV is unstable and can revert to neurovirulence in some instances.

Further investigation to determine whether the Sabin 1 poliovirus isolated was a normal Sabin or a VDPV have shown that the virus is a normal Sabin 1 poliovirus and not VDPV. It was very expedient to have screened for the presence of VDPV because of its latest implication in cases of acute flaccid paralysis in many parts of the world. Screening for Chikungunya virus using the multi-virus real time PCR did not also establish the aetiological agent. In West-Africa, similar clinical presentations of fever, convulsion, maculopapular rash and meningoencephalitis have been recorded to be caused by Chikungunya virus (CHIKV). Since its discovery in Tanganyika (Tanzania), Africa, in 1952, Chikungunya virus outbreaks have occurred sporadically in Africa, West Africa (Senegal and Nigeria), South Asia, and Southeast Asia with recent outbreaks spreading the disease over a wider range.

The study limitations include our inability to collect stool samples or anal swap since enteroviruses are readily present in stool. Secondly we did not investigate other DNA viruses with similar presentation. The etiological agent may be a DNA virus while the study was geared towards the detection of RNA virus. In the literature, other viruses associated with such manifestation are influenza, parainfluenza virus, herpes virus 6, Herpes virus 7 and RSV. Similar manifestations must be investigated for the presence of these viruses. Solving this puzzle will save the Government a lot of money that is spent on antibiotics, antimalarial and other drugs which in future will lead to drug resistance in the children.

## Conclusion

The findings from this study indicate that the aetiological agent for the observed convulsions in the children was neither due to non-polio enteroviruses nor chikugunya. We recommend that other viral agents capable of causing convulsion in children be investigated. The use of drugs to manage convulsions should be minimized and efforts should be made toward identifying the aetiological agent.
